# Cat-rodent *Toxoplasma gondii* Type II-variant circulation and limited genetic diversity on the Island of Fernando de Noronha, Brazil

**DOI:** 10.1186/s13071-017-2150-4

**Published:** 2017-05-03

**Authors:** Jean Carlos Ramos Silva, Fernando Ferreira, Ricardo Augusto Dias, Daniel Ajzenberg, Maria Fernanda Vianna Marvulo, Fernando Jorge Rodrigues Magalhães, Carlos Diógenes Ferreira Lima Filho, Solange Oliveira, Herbert Sousa Soares, Thais Ferreira Feitosa, Juliana Aizawa, Leucio Câmara Alves, Rinaldo Aparecido Mota, Jitender Prakask Dubey, Solange Maria Gennari, Hilda Fátima Jesus Pena

**Affiliations:** 1 Departamento de Medicina Veterinária, Universidade Federal Rural de Pernambuco, Rua Dom Manoel de Medeiros, s/n, Dois Irmãos, Recife, PE 52171-900 Brazil; 2 Departamento de Medicina Veterinária Preventiva e Saúde Animal, Faculdade de Medicina Veterinária Preventiva e Saúde Animal, Universidade de São Paulo, Av. Prof. Dr. Orlando Marques de Paiva, 87, Cidade Universitária, São Paulo, SP 05508-270 Brazil; 3 Instituto Brasileiro para Medicina da Conservação - Tríade, Rua Silveira Lobo, 32, caixa postal 48, Casa Forte, Recife, PE 52061-030 Brazil; 4 INSERM, Univ. Limoges, CHU Limoges, UMR_S 1094, Tropical Neuroepidemiology, Institute of Neuroepidemiology and Tropical Neurology, F-87000 Limoges, France; 5 Faculdade Max Planck, Rodovia João Ceccon, 60, Altos da Bela Vista, Indaiatuba, SP 13331-400 Brazil; 6 Universidade Paulista, Av. Comendador Enzo Ferrari, 280, Swift, Campinas, SP 13043-900 Brazil; 7 Administração do Distrito Estadual de Fernando de Noronha, Rua Dona Maria César, 68, Recife Antigo, Recife, PE 50030-140 Brazil; 8 Departamento de Medicina Veterinária, Instituto Federal da Paraíba, Sousa, PB 58800-970 Brazil; 9 Animal Parasitic Diseases Laboratory, Beltsville Agricultural Research Center, United States Department of Agriculture, Beltsville, MD 20705-2350 USA

**Keywords:** Toxoplasmosis, Feline, Synanthropic rats, Isolation, Microsatellite markers, PCR-RFLP markers

## Abstract

**Background:**

In Brazil, studies on animals and humans in mainland areas have shown that most strains of *Toxoplasma gondii* are pathogenic to mice and exhibit great genetic variability.

**Results:**

In this study, using a set of 11 PCR-RFLP and 15 microsatellite markers, we isolated and genetically characterised *T. gondii* strains from one cat and three rats on Fernando de Noronha Island. The cat had antibodies to *T. gondii*, which were revealed using a modified agglutination test (MAT, cut-off 1:25) and the seroprevalence among the 46 rodents was 15.2%. Viable *T. gondii* was isolated from one cat (TgCatBrFN1), two brown rats (TgRatnoBrFN1 and TgRatnoBrFN2) and one black rat (TgRatraBrFN1). Unlike the strains from mainland Brazil, these isolates were not pathogenic to outbred mice. The genotypes of these strains were compared with strains previously isolated on the island and in mainland Brazil. The analysis based on microsatellite data showed a limited genetic diversity of *T. gondii* on Fernando de Noronha Island with the majority of strains clustered into the following three groups: type II, III, and Caribbean 1.

**Conclusions:**

There was little variation among strains within the same group, suggesting that the majority of strains circulating on Fernando de Noronha are derived from only a few strains that were recently introduced to the island, likely from imported cats. Except for the strain belonging to the Caribbean 1 group that originates from northeast Brazil, there was little evidence that strains from the other groups were introduced to Fernando de Noronha *via* mainland Brazil.

**Electronic supplementary material:**

The online version of this article (doi:10.1186/s13071-017-2150-4) contains supplementary material, which is available to authorized users.

## Background

*Toxoplasma gondii* infections are prevalent in animals and humans on a global level [1]. Felids are key species in the life-cycle of *T. gondii* because sexual reproduction in their intestines leads to the production of millions of highly resistant oocysts [2]. The seroprevalence of *T. gondii* among humans is high in Brazil, reaching 90% in some regions, and may be related to high environmental contamination by oocysts [3]. The worldwide genetic diversity of *T. gondii* isolates has been studied extensively over the last two decades, showing that the hotspot of diversity is located in South America, particularly in Brazil [4]. Severe cases of ocular and congenital toxoplasmosis in Brazil have been associated with this high diversity [5].

Fernando de Noronha (3°50'28.9''S, 32°24'39.4''W) is an archipelago of 21 islands and islets in the Atlantic Ocean and is located approximately 354 km east of the Brazilian coast. The main island has a population of approximately 3,000 inhabitants [6]. There are no records regarding the juncture of when cats and rats were introduced to the island, but these animals may have come from Europe on ships that arrived on the archipelago, starting at the time of its first human occupation during the sixteenth century.

Previous studies reported high rates of *T. gondii* seroprevalence in different animal species in Fernando de Noronha, including cats, suggesting that transmission of this zoonotic parasite is active among the animal fauna of this Brazilian archipelago [7–9]. *Toxoplasma gondii* strains isolated from chickens, cats, and cattle egrets on Fernando de Noronha showed unexpected genotyping results. These results showed the presence of atypical genotypes that seemed endemic to the island as well as clonal type II strains that are common in Europe and North America but are virtually absent in mainland Brazil [3, 9, 10]. The objective of this study was to isolate and characterise *T. gondii* strains from one cat and several rodents on Fernando de Noronha with PCR-FLP and microsatellite markers and examine their genetic relationships with other strains previously isolated on the island and in mainland Brazil.

## Methods

### Sample collection

On October 5, 2013, an adult male cat with a terminal condition was admitted by its owner to the Animal Surveillance Centre (NVA) of the State District Administration of Fernando de Noronha in the village of Vila do Sueste. The cat was necropsied soon after death, and the heart, brain and thigh muscle samples were collected. A blood sample was obtained from the cardiac cavity and was centrifuged at 1,500× *g* for 10 min; the serum sample was stored at -20 °C.

Between October 2013 and April 2014, as part of a synanthropic rodent control programme on the Island of Fernando de Noronha, 28 black rats (*Rattus rattus*) and 18 brown rats (*Rattus norvegicus*) were captured with Tomahawk live traps at different locations on the island. The rodents were anaesthetised using ketamine hydrochloride (40–90 mg/kg) and xylazine hydrochloride (2–5 mg/kg) and blood samples (3 ml) were collected by means of cardiac puncture. The rodents were individually sacrificed in a hermetically sealed box containing cotton wool soaked with isoflurane. Necropsies were performed to collect brain, heart, and skeletal muscle samples from each rodent.

All cat and rodent samples were transported under refrigeration by air to the Faculty of Veterinary Medicine of University of São Paulo (FMVZ-USP) for laboratory analyses.

### Serological examination and bioassay

The serum samples from the cat and black and brown rats were tested using the modified agglutination test (MAT) [11], with a 1:25 dilution as the cut-off [12].

To conduct the bioassay, brain, heart and skeletal muscle samples from each rodent were homogenised together in a blender with 0.85% NaCl, whereas each cat tissue sample was homogenised separately. The homogenates from each animal were digested with an acidic pepsin solution [13] and subcutaneously inoculated into five or three outbred Swiss mice (1.0 ml per mouse). The surviving mice were bled six weeks post-inoculation (p.i.), and a 1:25 dilution of the serum from each mouse was tested for *T. gondii* antibodies with MAT. Mice were euthanised (using the same protocol as above) two months p.i., and their brains were examined for *T. gondii* tissue cysts [1].

### Genotyping analyses and neighbour-joining clustering

DNA samples from *T. gondii* strains collected in this study were extracted from mouse brains with the Qiagen® Dneasy® Blood & Tissue kit and genotyped using 11 PCR-RFLP and 15 microsatellite markers distributed on the 14 chromosomes and plastid of *T. gondii*, as described previously [14–16]. As shown in Additional file 1: Table S1, for comparison, we included the genotyping data of 24 strains previously collected on Fernando de Noronha Island [3, 10] and 41 strains isolated from two different regions of mainland Brazil described in other studies (14 strains from northeast Brazil [10, 17–20] and 27 from São Paulo [15, 21]). The ENT, ME49, and NED strains were used as reference *T. gondii* type I, II, and III strains, respectively [22].

Because microsatellite markers are better markers than PCR-RFLP for discriminating related isolates at a limited geographic scale such as an island [14], the neighbour-joining tree was only reconstructed from microsatellite data. The unrooted tree was reconstructed with Populations 1.2.32 (http://bioinformatics.org/populations/) based on the chord-distance *Dc* evaluation of Cavalli-Sforza and Edwards [23] and generated with MEGA version 6.05 [24] (http://www.megasoftware.net/history.php).

## Results

**Fig. 1:**
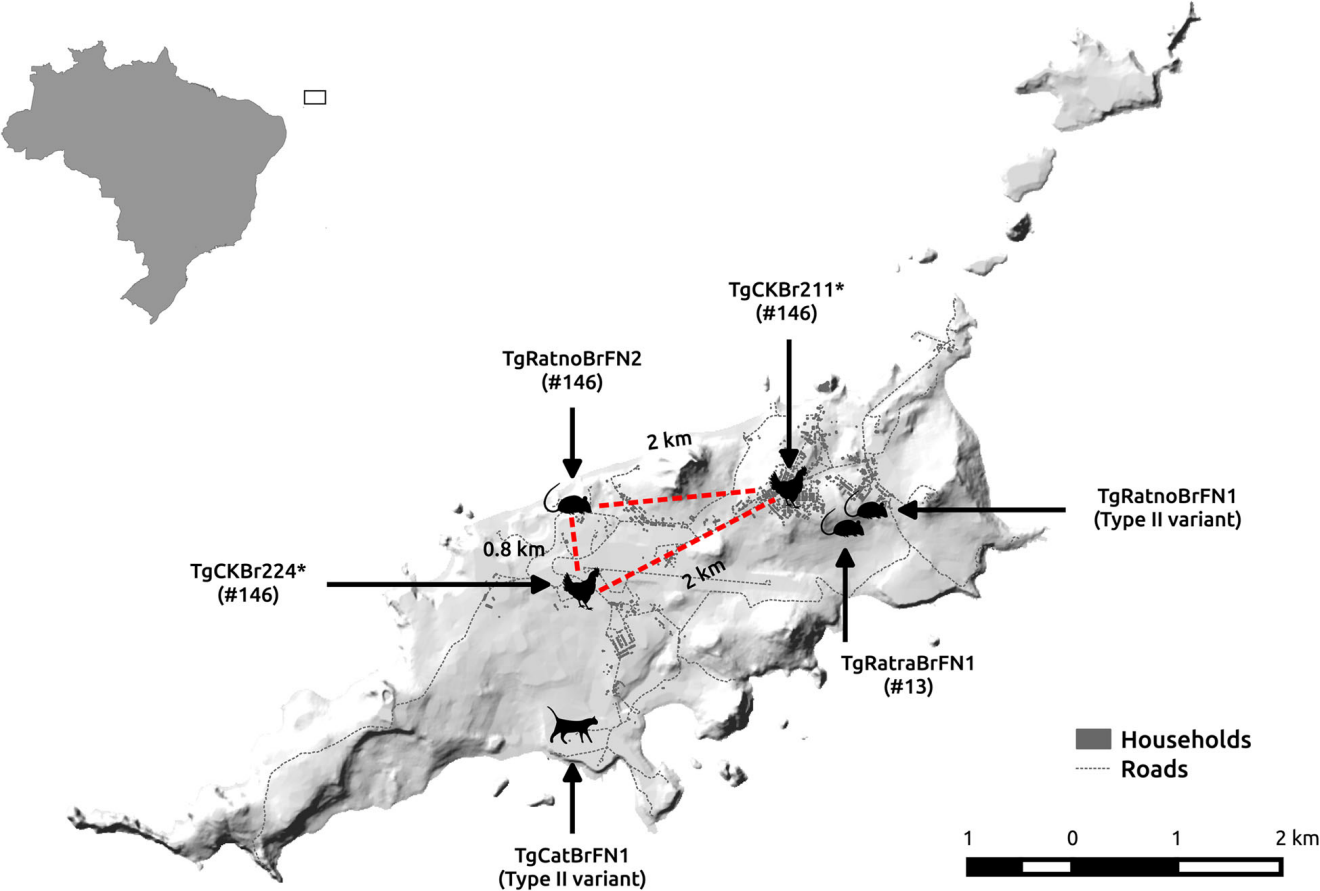
Map of Fernando de Noronha Island, Brazil, showing the locations where *Toxoplasma gondii* isolates were obtained. The asterisk indicates *T. gondii* isolates from chickens genotyped previously [3]

Antibodies to *T. gondii* were found in the cat, in five out of 28 black rats (18%), and in two out of 18 brown rats (11%). The MAT titres were as follows: < 25 (39 rodents), 25 (one black rat), 100 (cat), 800 (two black rats), 1,600 (two black rats and one brown rat), and 6,400 (one brown rat) (Table 1). Viable *T. gondii* strains were isolated from the cat, two brown rats and one black rat, and the isolates were named TgCatBrFN1, TgRatnoBrFN1, TgRatnoBrFN2 and TgRatraBrFN1, respectively (Fig. 1). None of the isolates were pathogenic in mice (Table 1).

**Table 1:** Isolation of *Toxoplasma gondii* from black and brown rats and antibody titers by Modified Agglutination. Test (MAT), Fernando de Noronha Island, Brazil

MAT titre	No. of rodents	No. bioassayed	No. of isolates	Mice bioassay^a^	*T. gondii* isolate ID	Rodent ID/species
				No. death/no. infected		
< 25	39	39	0	na	na	na
25	1	1	0	na	na	na
800	2	2	1	0/5	TgRatraBrFN1	R14/*Rattus rattus*
1,600	3	3	1	0/3	TgRatnoBrFN1	R38/*Rattus norvegicus*
6,400	1	1	1	0/3	TgRatnoBrFN2	R41/*Rattus norvegicus*
Total	46	46	3	0/11		

*Abbreviation: na* not applicable

^a^Parasite numbers were not evaluated in the inoculums

**Fig. 2:**
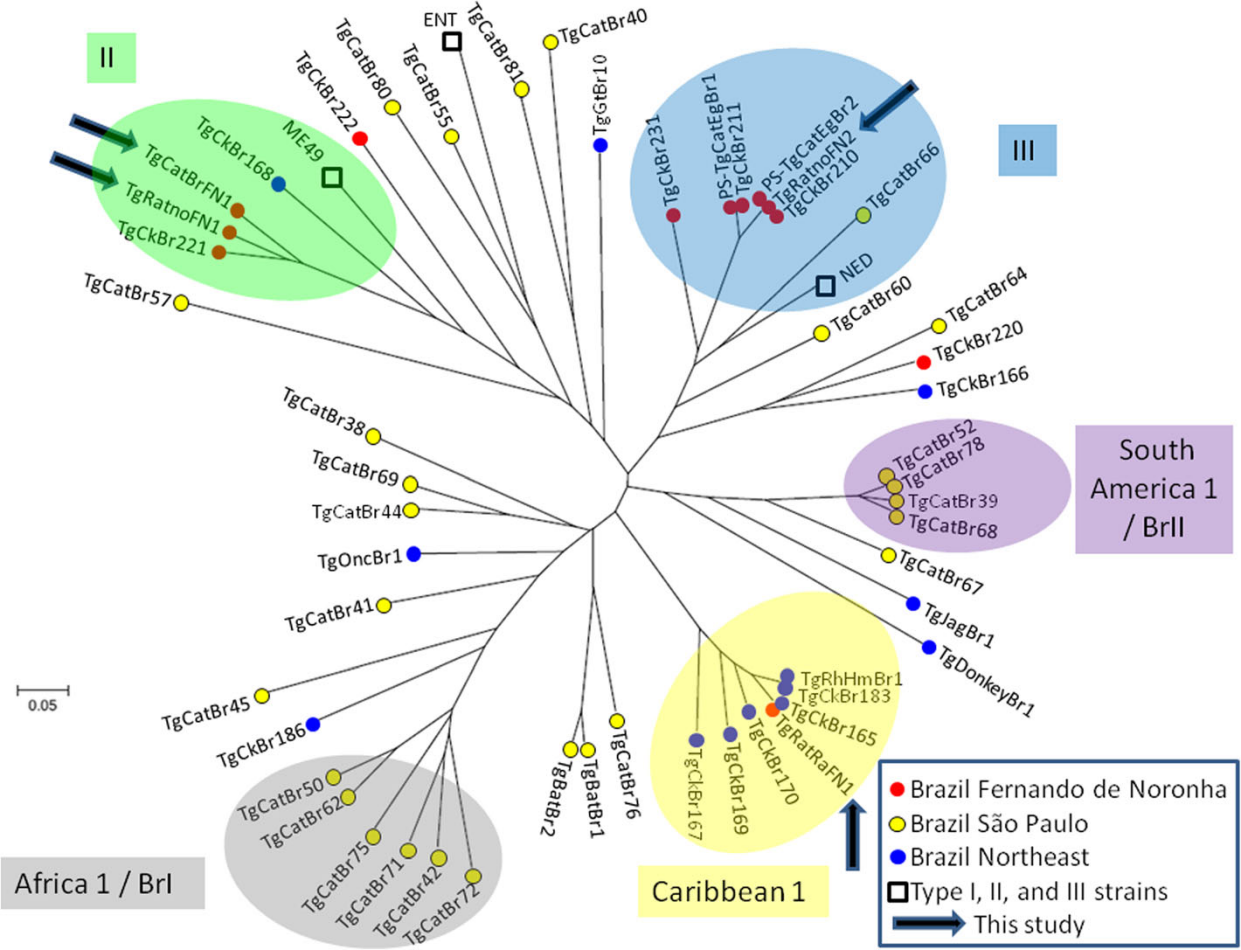
Neighbour-joining clustering of *Toxoplasma gondii* strains based on 15 microsatellite markers. Squares indicate type I (ENT), II (ME49) and III (NED) reference strains; red points indicate strains from Fernando de Noronha Island, yellow points indicate strains from São Paulo State; blue points indicate strains from northeast Brazil; arrows indicate the four strains collected in the present study. Strains TgCkBr221, 225, 226, 228 and 230 had 15 identical microsatellite markers and were represented by the TgCkBr221 strain in the tree (see Additional file 1: Table S1 for genotyping details); TgCkBr211, TgCkBr183 and TgCkBr210 were identical to TgCkBr224, TgCkBr184 and TgCkBr212-219, 223, 227 and 229, respectively

The genotyping results with RFLP and microsatellite markers are presented in Additional file 1: Table S1. The neighbour-joining analysis based on microsatellite data showed that all *T. gondii* strains but one (TgCkBr220) from Fernando de Noronha Island were highly structured in three groups: three strains (including TgCatBrFN1 and TgRatnoBrFN1 from this study) were clustered with the ME49 strain in the type II group, six strains (including TgRatnoBrFN2 from this study) were clustered with the NED strain in the type III group, and the TgRatRaBrFN1 strain was grouped in the Caribbean 1 group (Fig. 2). Inside each group, differences among strains were limited even with polymorphic microsatellite markers. For example, only one microsatellite marker was able to differentiate strains with the ToxoDB-RFLP genotype #146 in the type III group, even though they were collected from different hosts and at different times and locations on the island (Additional file 1: Table S1). Strains with the ToxoDB-RFLP genotype #3 that belong to the type II group were also very similar, with only three microsatellite markers able to differentiate them, and had the allele 329 at the microsatellite marker *N83* (Additional file 1: Table S1). This latter allele is absent in strains from Europe and North America and, to date, has been identified only in Brazilian strains (D. Ajzenberg, personal communication). Strains from common clonal lineages in mainland Brazil (BrI and BrII) were not identified in Fernando de Noronha.

## Discussion

The Island of Fernando de Noronha provides a unique opportunity to study the epidemiology of toxoplasmosis because of restricted animal and human populations. There is a high density of cats (*Felis catus*) in Fernando de Noronha: of the estimated 848 domestic animals living on the island, 470 are domestic cats [25]. We are not aware of the presence of any wild felid on the island. The number of cats seems to be more important than just their presence because as the number of cats increases, the number of oocysts in the environment increases [1, 26]. Since rats are considered an important food source for cats, we surveyed *T. gondii* infection in these intermediate hosts. All the rats were bioassayed, irrespective of serological findings, because congenitally infected rats may develop immune tolerance without antibody production [27]. *Toxoplasma gondii* antibodies were found in 15% of the 46 rats collected in our study, which is lower than the 38% [7] of black rats from Fernando de Noronha with the same test and cut-off; these data confirm that rats are significantly infected with *T. gondii* and contribute to the cycle of this zoonosis on the island.

Our results confirm the limited genetic diversity of *T. gondii* strains on the island. The minor variation between strains in the same group with polymorphic microsatellite markers suggests that the majority of *T. gondii* strains from Fernando de Noronha originated from a few strains that were recently introduced onto the island, likely from imported cats or possibly migratory birds. The TgRatraBrFN1 strain belongs to the Caribbean 1 group that is endemic to the Caribbean and the anthropised coast of French Guiana [28, 29]. In this study, we show that this Caribbean 1 group is also endemic to northeast Brazil, where the TgRatraBrFN1 strain likely originates. The ToxoDB-RFLP genotype #146 seems to be the dominant genotype on the island since it has been isolated from chickens, cats, rats, and even cattle egrets living on the island in prior studies [3, 9, 10] and this study. The neighbour-joining analysis with microsatellite markers indicates that this genotype is related to clonal type III strains, but the fact that this genotype has only been sampled to date on the island [4] and its significant divergence from type III strains with RFLP markers suggests a specific evolutionary history for this genotype. Type II strains have now been isolated from chickens, cats and rats from Fernando de Noronha, which confirms that the most successful clonal lineage from Europe and North America is circulating in Fernando de Noronha. The origin of this genotype on the island remains enigmatic because it is virtually absent from mainland Brazil except in the southernmost part of the country near the Argentinean and Uruguayan borders [30], and the presence of allele 129 at the microsatellite marker N83 excludes importation from Europe or North America.

## Conclusions

The State District Administration of Fernando de Noronha (ADEFN) has a programme to promote public health and control synanthropic animals. Our results support the implementation of a toxoplasmosis prevention and control programme based on the guidelines of One Health and Ecological Health (conservation medicine) in the archipelago of Fernando de Noronha.

## Additional file

Additional file 1: Table S1.
*Toxoplasma gondii* genotyping results with RFLP and microsatellite markers from strains from Fernando de Noronha Island and mainland Brazil. (XLSX 30 kb)

## Abbreviations

ADEFN: State District Administration of Fernando de Noronha
FMVZ-USP: Faculty of Veterinary Medicine of University of São Paulo
MAT: Modified agglutination test
NVA: Animal surveillance centre
PCR-RFLP: Polymerase chain reaction - restriction fragment length polymorphism
